# Kyste épidermoïde extradural géant

**DOI:** 10.11604/pamj.2019.33.301.19204

**Published:** 2019-08-19

**Authors:** Taoufik Africha, Omar Boulahroud

**Affiliations:** 1 Service de Radiologie, Hôpital Militaire My Ismail, Meknès, Maroc; 2 Service de Neurochirurgie, Hôpital Militaire My Ismail, Meknès, Maroc

**Keywords:** Kyste épidermoïde, extradural, IRM, Epidermoid cyst, extradural, MRI

## Abstract

We here report the case of a 68-year old female patient presenting with a 6-month history of headaches and difficulty walking with cerebellar syndrome and balance disorders. Brain CT scan showed voluminous extradural median lesion in the Posterior Cranial Fossa (PCF) with calcifications, erosion in the occipital bone and peripheral contrast enhancement. MRI for characterizing lesions showed lesion extradural median process of PCF with hyposignal T1 (A), hypersignal T2 (B), Flair heterogeneous signal (C) with moderate peripheric enhancement (D) and diffuse hypersignal. Surgical treatment allowed complete tumor resection and histological examination confirmed the diagnosis of epidermoid cyst. Epidermoid cysts of the PCF are rare, they are slow-growing congenital tumors but they can occur at any age, without sex predominance. The most common intradural lesions occur at the level of the cerebellopontine and parasellar angle. CT scan shows well-defined tumor process without contrast enhancement. MRI allows for better characterization of the lesion as in our case. Differential diagnosis is made with arachnoid and dermoid cysts. Treatment of epidermoid cysts is based on surgery with complete removal of the tumor and the capsule.

## Image en médecine

Une patiente de 68 ans qui consulte pour des céphalées et des troubles de la marche depuis 6 mois, avec un syndrome cérébelleux et des troubles de l'équilibre. Une tomodensitométrie cérébrale a montré une volumineuse lésion extra-durale médiane de la Fosse Cérébrale Postérieure (FCP) avec des calcifications, une érosion de l'os occipital et une prise de contraste périphérique. Une IRM de caractérisation a montré un processus lésionnel extradural médian de la FCP en hypo signal T1 (A), hyper signal T2 (B), hétérogène en Flair (C), avec rehaussement périphérique modéré (D), et hyper signal diffusion. Un traitement chirurgical a permis l'exérèse complète de la tumeur et l'examen histologique a confirmé le diagnostic de kyste épidermoïde. Les kystes épidermoïdes de la FCP sont rares, c'est des tumeurs congénitales à croissance lente mais peuvent se révéler à tout âge, sans prédominance de sexe. Les localisations intra-durales, au niveau de l'angle ponto-cérébelleux et para-sellaire, sont les plus habituelles. La tomodensitométrie montre un processus tumoral bien limité sans prise de contraste, c'est l'IRM qui permet une meilleure caractérisation de la lésion comme dans notre cas. Le diagnostic différentiel se fait avec les kystes arachnoïdiens et les kystes dermoïdes. Le traitement des kystes épidermoïdes est chirurgical avec ablation complète de la tumeur et de la capsule.

**Figure 1:**
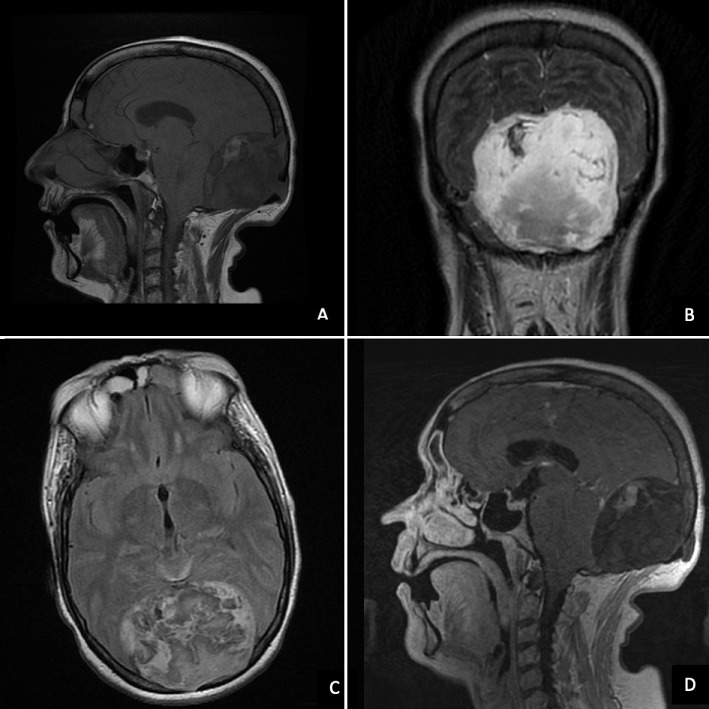
IRM cérébrale montrant un processus lésionnel extradural médian de la FCP en hyposignal T. (A) hypersignal T2, (B) hétérogène en FLAIR, (C) avec rehaussement périphérique modéré, (D) en rapport avec un kyste épidermoïde extra-axial

